# Is the Living Will an interesting way to determine themselves? Qualitative research about considerations said by oncologists in a university service care in Southeast Brazil

**DOI:** 10.1192/j.eurpsy.2022.1694

**Published:** 2022-09-01

**Authors:** E. Turato, J.R. Rodrigues, C. Santos, A.C. Bispo, C.S. Lima

**Affiliations:** State University of Campinas, Medical Psychology And Psychiatry, Campinas, Brazil

**Keywords:** Qualitative research, Living Will, defense mechanisms, Oncologists

## Abstract

**Introduction:**

What does the physician think in his/her intimacy about clinical care for when his/her death would be near? Living Will is a type of advance directive with the aim to guarantee the testator’s autonomy when faced with death. Particularly oncologists are often faced with human finitude. Their delicate work does not protect them from the possible anguish of thinking and preparing for their own death. It is pertinent to know the psychic mechanisms normally present in the management of this expectation.

**Objectives:**

To explore symbolic representations of oncologists such as referred to the possible elaboration of their own Living Will.

**Methods:**

Qualitative design. Eight participants, clinicians, sample closed by theoretical saturation of information. Semidirected interviews in-depth were conducted online during the pandemic, fully transcribed. Technique of Clinical-Qualitative Content Analysis used for data treatment to generate categories of discussion. The authors search for core meanings in the corpus of interviews, after free-floating readings.

**Results:**

Three categories emerged from the material: Living Will: postponing the decision in order to not anticipate death; From Rationalization Mechanism to Intellectualization: a more sophisticated defensive strategy; Loss of Autonomy: the doctor’s belief while to feel him/herself patient.

**Conclusions:**

(1) Even with all scientific knowledge, respondents have archaic thoughts on defining advance directives as healthy individuals would mean rushing time of their death. (2) Resistance of these professionals to an imagined scenario of end reveals underlying anguish in writing of living will. (3) There is fear of losing autonomy when they do not know how their Living Will can be seen.

**Disclosure:**

No significant relationships.

